# Early postoperative complications and discharge time in diabetic patients undergoing total shoulder arthroplasty

**DOI:** 10.1186/s13018-018-1051-3

**Published:** 2019-01-08

**Authors:** Brandon E. Lung, Michael Bisogno, Shrey Kanjiya, David E. Komatsu, Edward D. Wang

**Affiliations:** 1 School of Medicine, Stony Brook University, Stony Brook, NY USA; 2 Department of Orthopaedics, Stony Brook University Medical Center, HSC T-18, Room 080, Stony Brook, NY 11794-8181 USA

**Keywords:** Total shoulder arthroplasty, Diabetes mellitus, Insulin, Readmission, Postoperative complications, Outcomes

## Abstract

**Purpose:**

With the increasing elderly population and obesity epidemic, diabetes is an important factor in arthroplasty planning. Although research suggests diabetes is associated with increased postoperative morbidity after hip and knee replacement, the effect of diabetes and varying management with insulin versus non-insulin agents on total shoulder arthroplasty (TSA) is not established.

**Methods:**

All TSAs from 2015 to 2016 were queried from the American College of Surgeons National Surgical Quality Improvement Program database. Age, gender, BMI, steroid, ASA, operative time, and smoking status were compared between all diabetics, diabetics on insulin, diabetics on non-insulin agents, and non-diabetics to account for confounding variables. Thirty-day postoperative complications, readmission rate, surgical site infection (SSI), and non-routine discharge to rehabilitation were compared using bivariate and multivariate binary logistic regression. Postoperative time to discharge between diabetic groups was analyzed using univariate ANOVA with Tukey’s test.

**Results:**

The analysis included 7246 patients (insulin in 5% (*n* = 380), non-insulin in 13% (*n* = 922), and non-diabetics in 82% (*n* = 5944)). Diabetics were more likely to have an ASA ≥ 3 compared to non-diabetics (89.5% vs 50.1%; *p* < 0.001). Bivariate logistic regression showed statistical significance in readmission and non-routine discharge between all diabetics and non-diabetics (OR 1.7, 1.4; *p* = 0.001, 0.001), but there was no significance between SSI rate (0.3% vs 0.4%; *p* = 0.924). Multivariate logistic regression between groups showed significance in readmission between non-insulin diabetics vs non-diabetics (OR 1.5; *p* = 0.027), readmission and non-routine discharge in insulin vs non-diabetics (OR 2.1, 1.7; *p* = 0.003, < 0.001), and no significance between insulin and non-insulin diabetics. Postoperative days to discharge were 2.4, 2.0, and 1.8 days in insulin, non-insulin, and non-diabetics respectively. Mean differences were significant between all groups.

**Conclusions:**

Diabetic patients are at a higher risk for readmission and non-routine discharge compared to non-diabetics. Despite no increased risk in SSI, longer postoperative discharge time in diabetics should be considered in TSA planning.

**Trial registration:**

Not applicable

**Level of evidence:**

Level III, case-control study

## Background

With the increasing elderly population and rising obesity epidemic, diabetes mellitus is an important factor to consider in arthroplasty planning. The proportion of diabetic patients with functional disability is quickly increasing worldwide and is already becoming a major public health issue. Diabetic patients requiring surgical intervention remain a challenge to healthcare providers as suboptimal diabetic control has been associated with adverse perioperative outcomes such as infection, poor wound healing, metabolic complications, and increased mortality [1]. The prevalence of diabetes is projected to increase by 69% in developing countries and 20% in developed countries between 2010 and 2030 [2].

Taking into account age, disease duration, and vascular complications in a growing elderly population, the prevalence of musculoskeletal manifestations of type 1 and type 2 diabetes is as high as 30% of diabetic patients according to recent studies [3]. Yearly rates of total shoulder arthroplasty (TSA) are increasing at a higher rate than lower extremity arthroplasties with more than 50,000 TSAs performed per year in the USA. Increases in non-homebound discharge, length of stay, and total hospital charges in diabetics undergoing elective TSAs are important factors to consider in arthroplasty planning.

Although research suggests diabetes is associated with increased postoperative morbidity after hip and knee replacement, the effect of diabetes and varying management with insulin versus non-insulin agents on TSAs is not established. Diabetes has been speculated to damage rotator cuff tendons and impair bone healing through non-enzymatic glycosylation of collagen and chronic inflammation [4]. However, the literature regarding the impact of diabetes mellitus and HbA1C on upper limb arthroplasty outcomes is inconclusive and contradictory [5]. Statz et al. found no correlation between HbA1C and TSA complications, reoperations, or revisions [6]. Other studies report patients with diabetes are at increased risk for acute renal failure, longer hospital stays, infections, and increased non-homebound discharge [7–9]. Increase in blood sugar levels creates a glucose-rich environment leading to increased risk of surgical site infections (SSI) and delayed wound-healing [6]. Diabetic patients are frequently overweight and have higher perioperative risk of complications and cardiovascular risk factors that require close monitoring, patient self-care, and extensive financial resources.

The associations between poor bone quality and increased fracture risk in diabetics are barriers to increased physical activity in diabetic patients that ultimately lead to the increased need in joint arthroplasty. The aim of this study was to analyze the impact of diabetes and differences within the diabetics, characterized as non-insulin-dependent diabetes mellitus (NIDDM) and insulin-dependent diabetes mellitus (IDDM), on postoperative complications and discharge time in patients undergoing TSAs. Characterizing diabetes status additionally into NIDDM and IDDM may help healthcare providers consider further risk factors in not only diabetics but also relatively well-controlled (NIDDM) and poorly controlled diabetes (IDDM). Additional risk factors are important for both diabetic patients and surgeons to consider in elective TSA as poorer outcomes may influence decisions on treatment options.

## Materials and methods

All TSAs from 2015 to 2016 were queried from the American College of Surgeons National Surgical Quality Improvement Program (ACS-NSQIP) database. The study was exempt from approval by our University’s Institutional Review Board since all retrospective data included in the analysis were fully de-identified. This database contains de-identified surgical patient information from over 600 hospitals nationwide gathered by trained clinical reviewers collecting perioperative data on sites ranging from small community hospitals to large tertiary academic centers. During years 2015–2016, 274 collected variables were identified for surgical patients and included demographics, pre-operative lab values, operative time, intraoperative complications, and postoperative 30-day complications and readmission rates.

All patients who underwent TSAs were identified by Current Procedural Terminology code 23472, which included both anatomic and reverse TSAs. The study population consisted of all adults (≥ 18 years) undergoing TSAs between January 1, 2015, and December 31, 2016. Patients were classified as having no diabetes or diabetes mellitus requiring therapy with non-insulin agents or insulin. Patients not requiring any pharmacologic therapy or controlled by diet alone were considered part of the non-diabetic cohort. The NIDDM patients were considered to have a diagnosis of diabetes requiring therapy with a non-insulin anti-diabetic agent, such as oral agents, for at least > 2 weeks and currently on treatment within 30 days prior to surgery. IDDM patients were considered to have a diagnosis of diabetes requiring daily insulin therapy with or without concurrent non-insulin agent therapy.

Age, gender, BMI, ASA, operative time, long-term steroid use, and smoking status within the past year were compared between all diabetics, IDDM, NIDDM, and non-diabetics to account for baseline demographic characteristics. Patients with the regular administration of oral or parenteral corticosteroid or immunosuppressant medications within 30 days prior to surgery were categorized in the steroid cohort. Patients who smoked cigarettes (not including cigars, pipes, marijuana, or electronic cigarettes) at any point within the past year prior to admission for surgery were part of the smoking cohort.

Thirty-day postoperative complications analyzed included readmission rate, surgical site infections, myocardial infarction, pulmonary embolism, postoperative pneumonia, postoperative renal failure, postoperative bleeding requiring red blood cell transfusion, and non-routine discharge were compared using bivariate and multivariate binary logistic regression. Hospital discharge destination was dichotomized to routine (home) or non-routine, which included skilled care, unskilled facility, acute care, or rehabilitation. Normal distribution of the patients was assumed due to the large sample size.

Postoperative complications between diabetics (IDDM and NIDDM) and non-diabetics were compared in bivariate analysis by Pearson chi-square test for categorical data. Multivariable binary logistic regression analyses were used to determine whether diabetes status or differences in diabetic insulin therapy were independent risk factors for the abovementioned postoperative complications. Multivariable regression analyses were adjusted for age, gender, BMI, ASA, operative time, steroid use, and smoking status and reported as odds ratios in relation to the 95% confidence interval. Postoperative time to discharge between diabetic groups was analyzed using univariate ANOVA with Tukey’s test for multiple comparisons. Statistical analysis with *p* < 0.05 was performed using SPSS Software version 23.0 (SPSS, Chicago, IL, USA).

## Results

From 2015 to 2016, a total of 7246 patients undergoing TSAs were included in the study, including non-diabetics in 82% (*n* = 5944), NIDDM in 13% (*n* = 922), and IDDM in 5% (*n* = 380) (Table 1). Compared to non-diabetics, diabetic patients were more likely to be older (68.8 ± 9.9 years vs 69.6 ± 8.6 years; *p* = 0.003), have a higher BMI (30.5 ± 6.7 vs 34.3 ± 7.2; *p* < 0.001), and assigned ASA ≥ 3 (*p* < 0.001) with average ASA 2.8 in diabetics and 2.5 in non-diabetics. Differences in gender, smoking status, operation time, and steroid use between the two groups were not statistically significant. Among patients with diabetes mellitus, IDDM tended to have a higher average BMI (35.8 vs 33.7; *p* < 0.001), proportion of patients with ASA ≥ 3 (*p* < 0.001), and average ASA (3.0 vs 2.8; *p* < 0.001) compared to NIDDM. Compared to non-diabetics, NIDDM tended to also have a higher average BMI and ASA (*p* < 0.001).

**Table 1:** Baseline characteristics of all TSA patients and comparisons between diabetic groups from 2015 to 2016

Baseline characteristics	Non-DM (*n* = 5944)	NIDDM (*n* = 922)	IDDM (*n* = 380)	
Percentage of patients	82	12.7	5.2	
Average age	68.8	70.1	68.5	
Female gender %	56.4	53.6	54.5	
Average BMI	30.5	33.7	35.8	
Smoking %	11.3	10.2	9.5	
Steroid/immunosuppresant use %	5.1	5	3.4	
ASA ≥ 3%	50.1	77.3	89.5	
ASA	2.5	2.8	3	
Operation time (minutes)	108.52	109.58	110.61	
	Comparisons between diabetic groups			
Baseline characteristics	DM vs non-DM	Non-DM vs NIDDM	Non-DM vs IDDM	NIDDM vs IDDM
	*P* values			
Average age	*0.003*	*0.001*	0.771	*0.016*
Female gender %	0.089	0.105	0.457	0.769
Average BMI	*< 0.001*	*< 0.001*	*< 0.001*	*< 0.001*
Smoking %	0.185	0.341	0.285	0.693
Steroid/immunosuppresant use %	0.396	0.89	0.149	0.219
ASA ≥ 3%	*< 0.001*	*< 0.001*	*< 0.001*	*< 0.001*
ASA	*< 0.001*	*< 0.001*	*< 0.001*	*< 0.001*
Operation time (min)	0.303	0.766	0.632	0.92

*DM* diabetes mellitus, *NIDDM* non-insulin-dependent diabetes mellitus, *IDDM* insulin-dependent diabetes mellitus, *BMI* body mass index, *ASA* American Society of Anesthesiologists physical status classification system. *P* values ≤ 0.05 are in italics

In unadjusted bivariate analysis compared to non-diabetics, diabetic patients were more likely to encounter short-interval 30-day postoperative readmission (OR 1.7; *p* < 0.001) and non-routine discharge (OR 1.4; *p* < 0.001) (Table 2). Compared to non-diabetics, NIDDM were more likely to experience readmission (OR 1.6; *p* = 0.012), non-routine discharge (OR 1.3; *p* = 0.012), and postoperative transfusion due to bleeding (OR 1.8; *p* = 0.009). Unlike NIDDM, IDDM when compared to non-diabetics did not experience statistically significant difference in postoperative transfusion due to bleeding (*p* = 0.109). Between diabetics, there was no statistically significant difference in postoperative complications. There was no difference in SSI rates (0.3% vs 0.4%; *p* = 0.919), renal failure (*p* = 0.500), myocardial infarction (*p* = 0.813), pneumonia (*p* = 0.505), and pulmonary embolism (*p* = 0.540) between diabetic and non-diabetics.

**Table 2:** Unadjusted postoperative complications and comparisons between diabetic groups using binary logistic regression

	Non-DM (*n* = 5944) %	NIDDM (*n* = 922) %	IDDM (*n* = 380) %	DM vs non-DM *p* value	OR; 95% CI	
Readmission	2.7	4.2	5.5	*< 0.001*	1.724 (1.274–2.333)	
Non-routine discharge	10.5	13.1	15.2	*< 0.001*	1.386 (1.164–1.651)	
Postoperative SSI	0.4	0.3	0.5	0.919	0.951 (0.362–2.497)	
Postoperative renal failure	0.03	0.11	0	0.5	2.284 (0.207–25.203)	
Postoperative myocardial infarction	0.25	0.22	0.53	0.813	1.142 (0.381–3.421)	
Postoperative pneumonia	0.42	0.76	0.53	0.505	1.270 (0.629–2.566)	
Postoperative pulmonary embolism	0.32	0.33	0	0.54	0.684 (0.203–2.305)	
Postoperative transfusion due to bleeding	2.6	2.9	2.6	0.505	1.130 (0.788–1.620)	
	NIDDM vs non-DM *p* value	OR; 95% CI	IDDM vs non-DM *p* value	OR; 95% CI	NIDDM vs IDDM *p* value	OR; 95% CI
Readmission	*0.012*	1.58 (1.103–2.252)	*0.002*	2.088 (1.309–3.330)	0.312	0.755 (0.438–1.302)
Non-routine discharge	*0.012*	1.3 (1.059–1.596)	*0.001*	1.602 (1.206–2.128)	0.217	0.812 (0.583–1.130)
Postoperative SSI	0.724	0.805 (0.242–2.679)	0.718	1.305 (0.307–5.543)	0.598	0.617 (0.103–3.707)
Postoperative renal failure	0.339	3.226 (0.292–35.611)	0.995	0	0.994	0
Postoperative myocardial infarction	0.773	0.805 (0.185–3.509)	0.371	1.960 (0.449–8.557)	0.375	0.411 (0.058–2.927)
Postoperative pneumonia	0.194	1.741 (0.754–4.023)	0.801	1.204 (0.285–5.093)	0.647	1.446 (0.299–6.992)
Postoperative pulmonary embolism	0.957	0.967 (0.287–3.260)	0.994	0	0.994	5,273,569 (0)
Postoperative transfusion due to bleeding	*0.009*	1.759 (1.152–2.685)	0.109	1.674 (0.891–3.144)	0.891	1.051 (0.518–2.133)

*DM* diabetes mellitus, *NIDDM* non-insulin-dependent diabetes mellitus, *IDDM* insulin-dependent diabetes mellitus, *OR* odds ratio, *CI* confidence interval, *SSI* surgical site infection. *P* values ≤ 0.05 are in italics

**Fig. 1:**
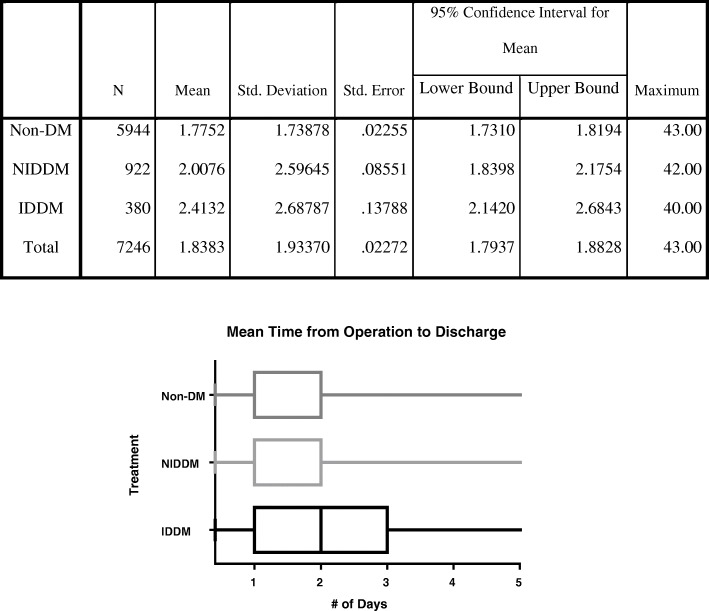
Mean time from operation to discharge and comparisons using univariate ANOVA with Tukey’s test. Non-DM, non-diabetes mellitus; NIDDM, non-insulin dependent diabetes mellitus; IDDM, insulin-dependent diabetes mellitus

In adjusted multivariable logistic regression compared to non-diabetics, diabetic patients were still more likely to encounter readmission (OR 1.7; *p* = 0.001) and non-routine discharge (OR 1.4; *p* = 0.001) (Table 3). After adjusting for baseline characteristics, NIDDM were not more likely to experience non-routine discharge (*p* = 0.058) and postoperative transfusion (*p* = 0.184) compared to non-diabetics. Compared to non-diabetics after adjusted regression, IDDM were still more likely to experience 30-day readmission (*p* = 0.003). Between diabetics, after adjusted regression, there was no statistically significant difference in postoperative complications. Time from operation to discharge in days was 2.4 days in IDDM, 2.0 days in NIDDM, and 1.8 days in non-diabetics (*p* < 0.001) (Fig. 1). Mean differences in discharge time were significant between all comparisons between non-diabetics, NIDDM, and IDDM.

**Table 3:** Adjusted postoperative complications and comparisons between diabetic groups using binary logistic regression

Postoperative complications	DM vs non-DM *p* value	OR; 95% CI
Readmission	*0.001*	1.661 (1.215–2.270)
Non-routine discharge	*0.001*	1.367 (1.132–1.650)
Postoperative SSI	0.924	0.953 (0.352–2.581)
Postoperative renal failure	0.384	1.187E+272 (0)
Postoperative myocardial infarction	0.943	1.442 (0.331–3.621)
Postoperative pneumonia	0.569	1.370 (0.229–2.966)
Postoperative pulmonary embolism	0.837	1.139 (0.330–3.933)
Postoperative transfusion due to bleeding	0.55	1.430 (0.388–1.920)
Postoperative complications	NIDDM vs non-DM *p* value	OR; 95% CI
Readmission	*0.027*	1.508 (1.049–2.169)
Non-routine discharge	0.058	1.235 (0.993–1.535)
Postoperative SSI	0.74	0.813 (0.239–2.766)
Postoperative renal failure	0.239	3.526 (0.792–31.611)
Postoperative myocardial infarction	0.53	1.811 (0.284–11.568)
Postoperative pneumonia	0.154	1.241 (0.954–5.023)
Postoperative pulmonary embolism	0.725	0.802 (0.235–2.741)
Postoperative transfusion due to bleeding	0.184	1.342 (0.869–2.072)
Postoperative complications	IDDM vs non-DM *p* value	OR; 95% CI
Readmission	*0.003*	2.065 (1.279–3.334)
Non-routine discharge	*< 0.001*	1.754 (1.291–2.384)
Postoperative SSI	0.734	1.293 (0.295–5.672)
Postoperative renal failure	0.995	0
Postoperative myocardial infarction	0.271	1.560 (.429–9.657)
Postoperative pneumonia	0.346	2.021 (.467–8.736)
Postoperative pulmonary embolism	0.994	0
Postoperative transfusion due to bleeding	0.856	0.941 (0.490–1.810)
Postoperative complications	NIDDM vs IDDM *p* value	OR; 95% CI
Readmission	0.261	0.731 (0.423–1.263)
Non-routine discharge	0.055	0.704 (0.496–1.276)
Postoperative SSI	0.613	0.629 (0.104–3.801)
Postoperative renal failure	0.994	0
Postoperative myocardial infarction	0.141	0.170 (0.016–1.794)
Postoperative pneumonia	0.809	1.216 (0.250–5.919)
Postoperative pulmonary embolism	0.994	5,167,031 (0)
Postoperative transfusion due to bleeding	0.337	1.426 (0.691–2.944)

*DM* diabetes mellitus, *NIDDM* non-insulin dependent diabetes mellitus, *IDDM* insulin-dependent diabetes mellitus, *OR* odds ratio, *CI* confidence interval, *SSI* surgical site infection. *P* values ≤ 0.05 are in italics

## Discussion

With the rise in the aging, overweight diabetic population likely to suffer from chronic musculoskeletal conditions, diabetic patients are susceptible to higher perioperative complications and longer hospital stays than non-diabetics [10, 11]. Although the role of diabetes on short-interval postoperative outcomes after lower extremity arthroplasty has been established, the effects of diabetes as an independent risk factor for adverse outcomes in TSAs remain unknown. Furthermore, the effects of daily insulin compared to non-insulin agents and no medical therapy on postoperative upper extremity arthroplasty outcomes have not been fully analyzed. In this large national cohort of 7246 TSA patients, the presence of diabetes mellitus is an independent risk factor for readmission, non-routine discharge disposition, and longer time from operation to discharge. While diabetes has been linked as a risk factor for SSI in orthopedic surgery, this study found no difference in SSI rates [12].

Among diabetic patients, insulin dependence predicted longer discharge time compared to non-insulin dependence, but there was no significant difference in postoperative complications. With the shift towards delivering cost-efficient care through bundled payments and outcomes-based reimbursements, the extended stay of greater than a half-day (0.6 days) between diabetics and non-diabetics is important to consider from a billing, hospital bed space, and hospital quality metrics perspective. Understanding the expected discharge disposition planning is important in our aging patient population with multiple comorbidities and is the initial step in being able to achieve interdisciplinary cost-effectiveness and better use of resources.

While diabetic patients tended to be older and have a higher BMI similar to previous studies of total hip and knee arthroplasty, unlike other studies, diabetics were not more likely to be females [13]. Although BMI has been associated with an increased risk of reoperation, mechanical failure, and superficial infection in shoulder arthroplasty, Garcia et al. found diabetes status to be the strongest independent predictor of postoperative complications compared to obesity and hypertension [14, 15]. In fact, BMI and age were adjusted for in the multivariate regression model, and the role of diabetes as an independent predictor of readmission in our study may reflect previous studies showing no difference in complication rates in TSA patients with BMI of 30–40 mg/kg [15]. Despite significant statistical significance in ASA status and proportion of ASA ≥ 3 among the diabetic groups, the average ASA were similar between non-diabetics (2.5) and diabetics (2.8), and ASA was adjusted for in the multivariate regression as a potential confounding variable. While diabetes has been shown to reduce intramembranous ossification, endochondral bone formation, and matrix synthesis, the chronic inflammatory state does not appear to affect operation time in this study [16].

Diabetes status was an independent predictor of 30-day readmission rate and is important to consider as the Diabetes Control and Complications Trial previously showed a 70% reduction in diabetic complications require advanced patient education, healthcare team collaboration, and extensive financial resources [17]. Reducing readmission rates may call for changes in healthcare practices and increased communication with patients on the importance of self-motivated preoperative health optimization. In fact, validated postoperative reverse TSA rehabilitation protocols are lacking, and Oliva et al. showed that personalized postoperative treatment protocols specific to patients stratified by perioperative risks can decrease complication rates [18]. High-risk patients treated with frequent physician consultations with established goals of recovery and adjusted rehabilitation protocols are shown to have improved clinical results and decreased complications [18]. As the landscape of healthcare economics shifts to value patient satisfaction outcomes, diabetes status is important to consider as part of a stratified rehabilitation protocol for a growing elderly population with inherent risk factors. Reverse TSA dislocation and complication rates are associated with poor healing of repaired soft tissues, and diabetes is important to take into account in the postoperative recovery phase as delayed growth factor production, endothelial progenitor cell dysfunction, and reduced granulation tissue formation are thought to impair wound and bone healing [18–20].

Although Ponce et al. found diabetics to be 1.5 times more likely than non-diabetics to have acute renal failure after shoulder arthroplasty, this study found no significant difference [7]. While unadjusted analysis showed NIDDM to have a greater likelihood of postoperative transfusion due to bleeding compared to non-diabetics, adjusted regression found no significant difference. In fact, King et al. found age, gender, and cardiovascular disease to be baseline characteristics predicting postoperative blood transfusion after TSA [21].

A diagnosis of diabetes has been associated with increased postorthopedic surgery inpatient mortality, morbidity, length of stay, and ICU admission [22]. In this study, the presence of diabetes, especially IDDM, predicted greater non-routine discharge disposition to rehabilitation or a form of enhanced recovery facility as opposed to routine home discharge. Non-routine discharge is important to consider as diabetic patients should be made aware of the risks of longer recovery times and unforeseen financial burdens associated with increased need for extensive rehabilitation. Similarly, increased time from operation to discharge among diabetic groups reflects poorer bone tensile strength, wound healing, and fracture risk suggested in previous diabetic animal models [23, 24]. Non-routine discharge and longer discharge times in diabetic patients suggest poorer return to independent mobility and activities of daily living with increased hospitalization costs.

While this study examines diabetes and insulin dependence as risk factors for poorer postoperative complications, other measurements of diabetes, such as HbA1C, have mixed associations according to the literature. Ekinci et al. found no association between HbA1C levels and length of stay, readmission rates, and complication rates in orthopedic surgical inpatients [25]. Similar to this study, Ekinci et al. observed no significant postoperative associations between diabetes and SSI, thromboembolism, and acute renal failure. Although Fink identified diabetes mellitus as a risk factor for shoulder arthroplasty infections, SSI rates among TSA patients did not differ by diabetes status in this study possibly due to overall low numbers of SSI reported in the database [26]. However, increased SSI awareness in postoperative diabetic TSA patients should still be taken into account as Statz et al. identified the glucose-rich surgical site environment and diminished systemic wound-healing capacity as ideal for thriving bacteria [6].

Despite the large number of patients included in this study, there are limitations to be considered in using the NSQIP database. Postoperative complication rates are limited to 30-day outcomes, and many end points, such as SSI, often occur beyond the 30-day follow-up period. Although the use of reverse total shoulder arthroplasty is becoming more prevalent, the use of Current Procedural Terminology coding 23472 to identify TSAs was not able to separate reverse from anatomical arthroplasty. However, previous studies have found both anatomic and reverse TSA to have similar complication rates, revision rates, and patient-reported outcomes, and this study focuses on short 30-day hospital quality metrics [27]. Although diabetes status was assigned by trained clinical reviewers, HbA1C and fasting glucose levels were not available to confirm level of glycemic control, and type 1 and type 2 diabetics were not able to be differentiated among the diabetics. However, the absence of distinction between type 1 and type 2 and complicated versus uncomplicated diabetics in this study is consistent with previous retrospective population-based arthroplasty studies since the low percentages of type 1 diabetics (0.20%) would increase the risk of finding false and misleading clinical results [28]. While this study suggests the role of diabetes as an independent risk factor for postoperative complications, this study does not establish causality and the odds ratios should be interpreted accordingly.

## Conclusions

Overall, this study provides evidence that diabetes status is an independent risk factor for postoperative readmission, non-routine discharge, and increased time to discharge. IDDM and NIDDM patients should be made aware of the increased risk of slower return to independent functional mobility. Communication between healthcare providers and diabetic patients is important in implementing precautionary measures to enhance postoperative recovery.

## Abbreviations

ACS-NSQIP: American College of Surgeons National Surgical Quality Improvement Program
DM: Diabetes mellitus
IDDM: Insulin-dependent diabetes mellitus
NIDDM: Non-insulin-dependent diabetes mellitus
SSI: Surgical site infection
TSA: Total shoulder arthroplasty
